# Long non-coding RNA SNHG1 promotes bladder cancer progression by upregulating EZH2 and repressing KLF2 transcription

**DOI:** 10.1016/j.clinsp.2022.100081

**Published:** 2022-09-07

**Authors:** Jie Min, Jiaxing Ma, Qi Wang, Dexin Yu

**Affiliations:** Department of Urology, The Second Hospital of Anhui Medical University, Hefei, Anhui, China

**Keywords:** Bladder cancer, Long non-coding RNA SNHG1, Competing endogenous RNA, miR-137-3p, EZH2, KLF2

## Abstract

**Objective:**

Long Non-Coding RNAs (LncRNAs) act as an indispensable role in cancer development. The study aimed to investigate the role and mechanism of lncRNA Small Nucleolar RNA Host Gene 1 (SNHG1) in Bladder Cancer (BC) progression.

**Method:**

The expression, prognostic value, diagnostic value, and correlation of SNHG1, Enhancer of Zeste 2 polycomb repressive complex 2 subunit (EZH2), and Kruppel Like Factor 2 (KLF2) were analyzed through bioinformatics analysis. The expression was also validated in BC tissues and cell lines. Besides, their regulation and binding were tested via qPCR, Western blot, Dual-Luciferase Reporter Assay (DLRA), Argonaute RISC catalytic component 2-RNA Immunoprecipitation (AGO2-RIP), and Chromatin Immunoprecipitation (ChIP). A xenograft model in nude mice was also established.

**Results:**

SNHG1 was significantly overexpressed in BC tissues and cells. Importantly, SNHG1 was associated with poor survival, and ROC curves revealed high diagnostic values. Moreover, by CCK8, wound healing, transwell, and Western blot analysis, SNHG1 knockdown significantly inhibited the proliferation, migration, invasion, and epithelial-mesenchymal transition of BC cells. Additionally, *in vivo* experiments showed that silencing SNHG1 hindered tumorigenesis and tumor growth. Regarding mechanism, the results of AGO2-RIP, ChIP or DLRA showed that SNHG1 played different roles at diverse subcellular sites. In the cytoplasm, SNHG1 acted as a competing endogenous RNA for miR-137-3p to promote EZH2 expression. In the nucleus, SNHG1 could interact with EZH2 to inhibit KLF2 transcription.

**Conclusion:**

Our study elucidated that SNHG1 formed a regulatory network and played an oncogenic role in BC, which provided a novel therapeutic target for BC treatment.

## Highlights

• lncRNA SNHG1 played an oncogenic role in bladder cancer, indicating poor prognosis.
• SNHG1 competitively bound miR-137-3p to promote EZH2 expression in the cytoplasm.
• SNHG1 could interact with EZH2 to inhibit KLF2 transcription in the nucleus.

## Introduction

Bladder Cancer (BC) is a malignant tumor that occurs in the mucosa of the bladder. The incidence rate of BC is ranked seventh worldwide.^1^^,^^2^ In China, BC is a common tumor of the urogenital system.^3^, ^4^, ^5^ BC occurs in all age groups from childhood to old age, especially in people over 60 years old.^6^ Smoking and occupational exposure are the two major factors for BC.^7^ 30% to 50% of patients are caused by smoking, which can increase the risk rate by 2 to 6 times. And long-time frequent smoking will lead to an obvious increase in the incidence.^8^ Occupational exposure accounts for about 25%, including coal tar, asphalt, dyes, and rubber.^9^

In the past, people did not focus on Non-Coding RNAs (ncRNAs), because they did not have the protein-coding potential.^10^ Today, more and more studies have demonstrated that ncRNAs have a variety of important functions in many diseases and cancers.^11^ Long Noncoding RNAs (lncRNAs) belong to a kind of ncRNAs. And lncRNAs are composed of more than 200 nucleotides, which have regulatory functions at the transcriptional and post-transcriptional levels.^12^ LncRNAs regulate chromatin structure in the nucleus and the stability of mRNAs in the cytoplasm.^13^, ^14^, ^15^ The abnormal expression of lncRNAs in clinical cases indicates that lncRNAs participate in the development of these diseases. LncRNAs participate in regulating cell proliferation, metastasis, and angiogenesis in tumor tissues.^11^^,^^16^ At present, it is still necessary to extensively investigate the functions and regulatory mechanisms of lncRNAs in BC. This may be beneficial for the treatment of BC patients.

LncRNA small nucleolar RNA Host Gene 1 (SNHG1) is widely distributed in the organism. SNHG1 gene is located on chromosome 11q12.3 and participates in various biological activities.^17^ SNHG1 expression was upregulated in hepatocellular carcinoma cells, which promoted tumor growth and inhibited apoptosis by activating Akt signaling pathway.^18^ SNHG1 led to abnormal transcription of genes by altering chromatin structure, thus promoting tumor growth.^17^^,^^19^ According to the report, SNHG1 was high-expressed in BC.^20^ However, the molecular mechanisms of SNHG1 in BC have not been well understood. In this study, the role of SNHG1 in BC was investigated, which provided a potential target for the diagnosis of BC.

In our study, it was found that SNHG1 played a vital role in BC. SNHG1 was significantly overexpressed in BC. And SNHG1 regulated BC cell proliferation, migration, invasion, Epithelial-Mesenchymal Transition (EMT), and tumorigenesis *in vitro* and *in vivo*. Mechanism studies found that SNHG1 played different roles in the cytoplasm and nucleus. And SNHG1 was involved in BC progression through regulating the Enhancer of Zeste Homolog 2 (EZH2) and Kruppel Like Factor (KLF2) expression.

## Materials and methods

### Bioinformatics analysis

The SNHG1 expression was analyzed in GSE7476 (9 tumor and 3 normal samples) and GSE65635 (8 tumor and 4 normal samples) from Gene Expression Omnibus (GEO) database. In level 3 HTSeq-FPKM (Fragments Per Kilobase per Million) format from the BLCA (Bladder Urothelial Carcinoma) of The Cancer Genome Atlas (TCGA) program, RNAseq data from 19 normal samples and 414 tumor samples were converted to Transcripts Per Million reads (TPM) format and log2-transformed. The corresponding clinical information was collected. SNHG1, EZH2, and KLF2 expressions in different groups were illustrated using ggplot2 (3.3.3) of R (3.6.3). Besides, EZH2 and KLF2 expressions were also analyzed in Oncomine database. The correlation of SNHG1, EZH2, and KLF2 expression in BLCA of TCGA was measured via Spearman analysis. The diagnostic value of SNHG1 for BLCA was tested by the pROC package (version 1.17.0.1). In terms of prognostic value, UALCAN database^21^ was utilized.

The prognostic value of miRNAs was extracted from the KMplotter database^22^ and plotted as a forest plot, patients were split by the median values of the gene/miRNA expression levels. The targets of hsa-miR-137-3p were predicted from TargetScan and miRDB databases and intersected with upregulated genes in BLCA in the GEPIA2 database. The overlapped genes were subjected to prognostic analysis in the GEPIA2 database^23^ (the significant gene was labeled with red square) and ROC analysis in the TCGA database.

### Clinical samples

For the research, clinical samples were provided by the Second Hospital of Anhui Medical University, including BC tissues in 21 cases receiving histological diagnosis and non-neoplastic adjacent tissues in 16 cases. All fresh and intact tissues were confirmed by histopathology. Specimens were labeled and stored at -80°C or in liquid nitrogen. The research program was approved by the ethics committee of the Second Hospital of Anhui Medical University, and all patients provided written informed consent.

### Cell culture

J827 cells (BC cell line) were purchased from the Shanghai Fusheng Industrial Co., Ltd. (Shanghai, China). 5637 BC cell line and SV-HUC1 cells (human normal epithelium of bladder) were acquired from the American Type Culture Collection (ATCC). J827 cells and 5637 cells were cultured in RPMI-1640 (Invitrogen). SV-HUC1 cells were cultured in F-12K (Gibco) with 10% Fetal Bovine Serum (FBS, Gibco). Culture conditions were carried out in an incubator with 5% CO_2_ and 95% air at 37°C.

### Quantitative-PCR (q-PCR) analysis

The PureLink RNA Mini assay kit (Thermo Fisher Scientific, USA) was used to harvest total RNA in cells and samples. The PrimeScript RT Reagent assay kit (Thermo Fisher Scientific, USA) was used to perform reverse transcription. A specific miRNA Reverse Transcription kit (Thermo Fisher Scientific, USA) was used for the reverse transcription of miRNA. The expressions of miRNA and target genes were standardized with reference to U6 or GAPDH. The Cytoplasmic & Nuclear RNA Purification Kit (Norgen Biotek, Germany) was used to respectively isolate the SNHG1 from the cytoplasm or Nucleus of 5637 cells and J827 cells. And the expression of SNHG1 was detected by q-PCR. The relative expression was calculated by using 2-ΔΔCt. The primer sequences of SNHG, EZH2, KLF2, and GAPDH were synthesized by Sangon Biotech (Shanghai, China). And the primers of miR-21-5p, miR-137-3p, miR-194-5p, miR-4735-3p, and U6 were synthesized from RiboBio (Guangzhou, China). All primer sequences are listed in Supplementary Table 1.

### Transfection of cell lines

The cells were inoculated in 6-well plates, and the cell fusion rate reached 60%‒80% the next day. miR-137-3p antagomir and mimics were obtained from Ribobio (Guangzhou, China). Sh-SNHG and sh-NC were synthesized by GenePharma (Hangzhou, China). The cDNA of SNHG was cloned into pcDNA3.1 (Invitrogen, USA). p-CMV-HA (Invitrogen, USA) was used to construct an overexpression complex of EZH2. Transfection reagent lipo 3000 and RNAiMAX (Invitrogen, USA) were applied for transfection experiments. All primer sequences are listed in Supplementary Table 2.

### CCK-8 assay

1 × 10^3^ cells/mL suspension was added to a 96-well plate. The cells were cultured separately for 24h, 48h, and 72h at 37°C. Then the cell viability was evaluated with CCK-8 reagent (Sigma, USA) at 10-μL/each well. After being incubated in an incubator for 1h, the absorbance of cells was measured at 450 nm by a spectrophotometer.

### Wound healing assay

Cells were uniformly inoculated into each well of a 6-well plate and incubated at high concentrations overnight. A wound line was scrapped on the cell layers with a sterile plastic pipette tip of 200 μL. The suspended cells were washed with PBS. The state of cell layers was photographed and recorded at 0h and 48h by a microscope, respectively. During this period, 6-well plates were incubated at 37°C in a 5% CO_2_ incubator.

### Transwell assay

Transwell chambers (Corning, USA) with membrane pores of 8-μm contained diluted Matrigel (BD Biosciences), and the cell invasion was determined. 4 × 10^5^ cells were seeded into the upper chamber with a serum-free medium. The bottom chamber contained a medium with 10% FBS. Then cells were incubated for 24h. 4% paraformaldehyde was used to fix cells. 1% crystal violet was used to stain cells. The number of cells was counted from 10 randomly selected regions.

### Western blot assay

RIPA lysis buffer (Fdbio science, China) was used to extract the total proteins from cells and samples. Protein samples were separated by 10% SDS-PAGE. Antibodies PCNA (1:1000, 131105, CST), E-cadherin (1:1000, 31955, CST), N-cadherin (1:1000, 40615, CST), Vimentin (1:1000, ab137321, Abcam), MMP-9 (1:1000, ab283575, Abcam), EZH2 (1:1500, ab186006, Abcam) and KLF2 (1:1000, ab194486, Abcam) were used to incubate PVDF membranes overnight at 4°C. The next day, secondary antibodies were diluted to incubate PVDF membranes for 1h. The blotted proteins were visualized by dropping an enhanced chemiluminescence solution, and these photographs were analyzed using Image J.

### RNA immunoprecipitation (RIP)

miR-137-3p mimics or NC mimics were transfected for 48 hours. Ago2 antibody (Abcam, UK), EZH2 antibody (Abcam, UK) and IgG (Abcam, UK) were used for RNA Immunoprecipitation (RIP) with the EZ-Magna RIP kit (Merck, Germany). qPCR assay was used to detect the enrichment of SNHG1 and EZH2.

### Chromatin immunoprecipitation assay

Transfection of J827 cells with sh-SNHG1 was performed. Chromatin immunoprecipitation (ChIP) experiment was carried out according to the detailed instructions of the ChIP Assay Kit (Upstate Biotechnology, USA). The lysates were immunoprecipitated with anti-EZH2 and anti-IgG. Immunoprecipitated DNA fractions were measured by qPCR.

### Dual-luciferase reporter assay

The mutation sites of SNHG1 and EZH2 fragments were designed for miR-137-3p. The vectors and mimics were transfected into BC cells. Dual-Luciferase Reporter Assay System (Promega, USA) was picked to measure the luciferase activity of wide-type and mutated genes. After sh-SNHG1 or p-CMV-HA-EZH2 transfection, KLF2 transcriptional activity was measured in J827 cells.

### Nude mouse models in vivo

The nude mice were purchased from Guangdong Medical Laboratory Animal Center (Guangdong, China) and were raised in a specific pathogen-free animal room (Guangzhou, China). Stably transfected J827 cells were collected and subcutaneously transplanted into the nude mice to create a tumor growth model. Tumor volumes were measured weekly and photographed. After 6 weeks, mice were subjected to general anesthesia with 1% pentobarbital sodium. Intact tumor tissues were obtained, and tumor size and weight were measured. Tumor tissues were dissected and placed in liquid nitrogen for future experiments. Every procedure was approved by the Animal Care and Use Committee of the Second Hospital of Anhui Medical University.

### Statistical analysis

Statistical analyses were performed using GraphPad Prism 7 (GraphPad Software, San Diego, CA, USA). First, the data were subjected to a normality test. One-way ANOVA with Bonferroni post-hoc test was used to analyze the differences in three groups or more groups. Two-tailed unpaired *t*-test was used to analyze the differences between the two groups. Total data were expressed as mean ± Standard Error of the Mean (SEM). And p < 0.05 was considered a statistically significant result.

## Results

### SNHG1 was overexpressed in BC and associated with poor prognostic outcomes

**Fig. 1:**
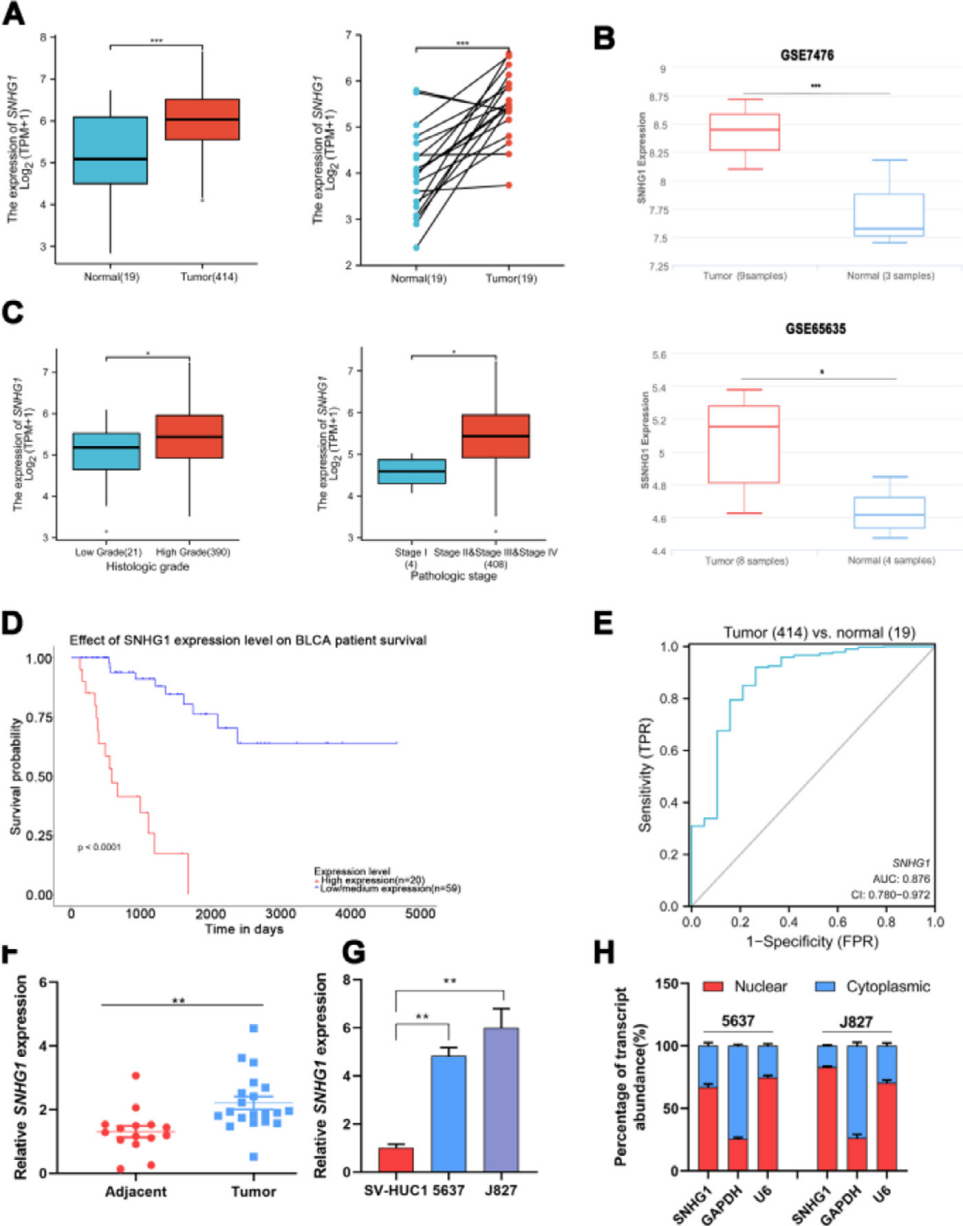
SNHG1 was highly expressed in BC. A, The expression of SNHG1 in BLCA data from TCGA (left) and fresh frozen specimen (right). B, SNHG1 expression in GSE7476 and GSE65635. C, SNHG1 expression in BLCA from TCGA database were shown in disparate histological subtypes or different stages. D, The prognostic value of SNHG1 was predicted by Kaplan-Meier analysis. E, The ROC curve analysis of SNHG1. F, SNHG1 expression in BC tissues and adjacent tissues. G, SNHG1 expression in BC cells and SV-HUC1 cells. H, The subcellular localization of SNHG1 in 5637 cells and J827 cells. Statistical significance was assessed using t test for two groups or one-way ANOVA for multiple groups. *p < 0.05; **p < 0.01; ***p < 0.001.

To investigate the effects of SNHG1 in BC, the authors firstly analyzed the expression of SNHG1 in Bladder Urothelial Carcinoma (BLCA) from TCGA. Among BC, BLCA is the most widespread and accounts for 90%.[1] The results showed that SNHG1 expression was elevated in BLCA compared to expression in non-neoplastic adjacent tumors (Fig. 1A). And the authors downloaded two datasets related to BC from GEO. Compared with normal samples, GSE7476 and GSE65635 displayed high expression of SNHG1 in tumor samples (Fig. 1B). In addition, SNHG1 was highly expressed in BC patients with high histological grade or high pathological stages (Fig. 1C). Then, Kaplan Meier was performed to analyze the correlation between the expression of SNHG1 and BC prognosis. It was indicated that BC patients with high SNHG1 expression had shorter survival compared to those with low/medium expression. (Fig. 1D). And ROC curve showed that the AUC of SNHG1 was 0.876 (Fig. 1E). Next, SNHG1 expression was measured in clinical tissues and cell lines of BC. Compared with adjacent tissues, the results of q-PCR showed high expression of SNHG1 in BC tissues (Fig. 1F). Similarly, compared with the normal epithelium of bladder SV-HUC1 cells, SNHG1 expression was higher in 5637 cells and J827 cells (Fig. 1G). In addition, in these two kinds of BC cells, the authors found that SNHG1 was distributed in the cytoplasm and nuclei, and it was more common in the nucleus (Fig. 1H). To sum up, SNHG1 was abnormally expressed in the development of BC.

### SNHG1 acted as a ceRNA to absorb miR-137-3p in the cytoplasm

**Fig. 2:**
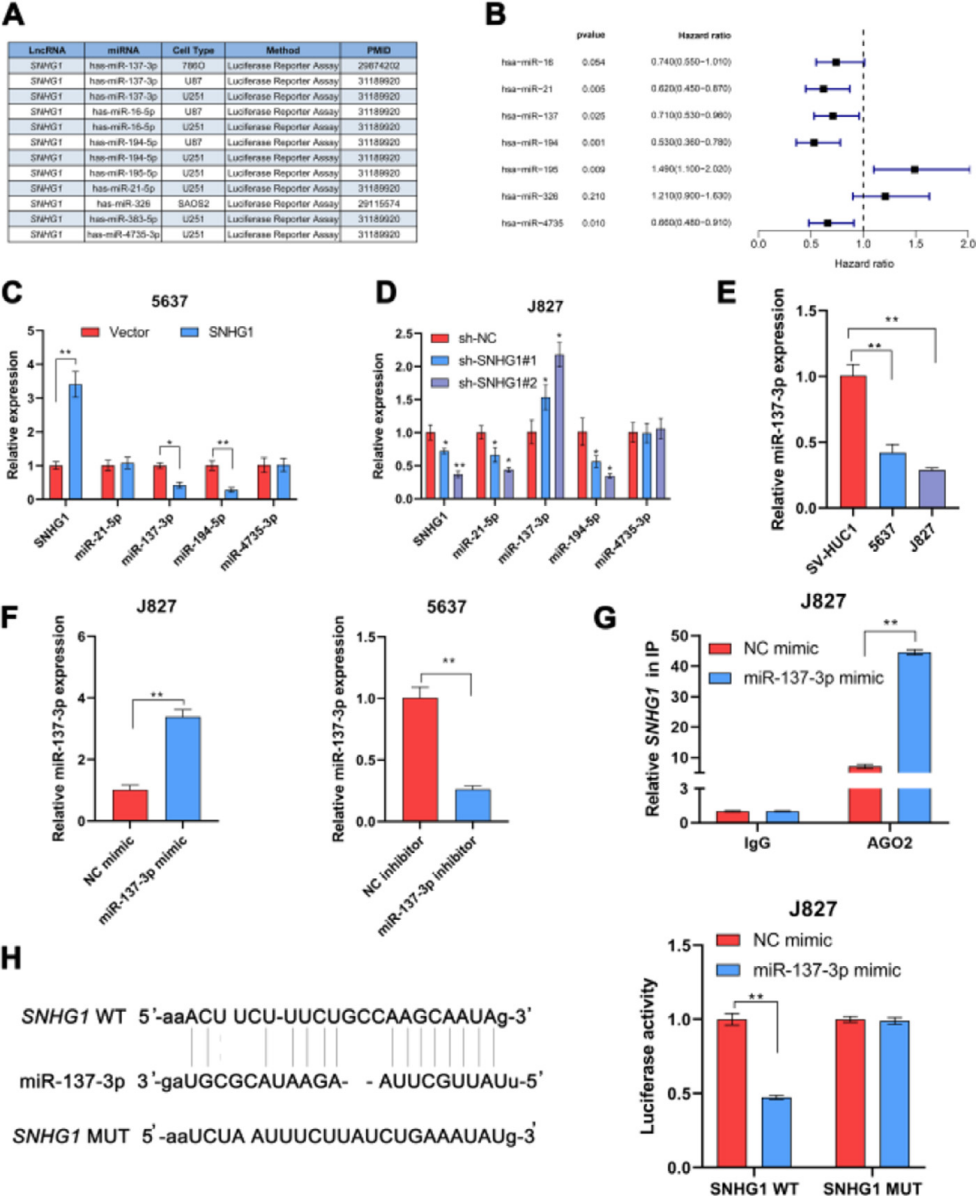
SNHG1 absorbed miR-137-3p in the cytoplasm. A, miRNAs reportedly absorbed on SNHG1. B, The forest plot of seven miRNAs following Kaplan-Meier analysis via KMplotter database. C, Expressions of four miRNAs in 5637 cells with SNHG1 overexpression. D, Expressions of four miRNAs in J827 cells with SNHG1 knockdown. E, miR-137-3p expression in BC cell lines and SV-HUC1 cells. F, miR-137-3p expression in J827 cells transfected with mimics or inhibitors. G, The enrichment of SNHG1 pulled down by Ago2 antibodies in J827 cells with mimic transfection. H, The luciferase activity in J827 cells co-transfected with SNHG1 plasmids and mimics was examined by dual luciferase reporter assay. Statistical significance was assessed using t test for two groups or one-way ANOVA for multiple groups. *p < 0.05; **p < 0.01. WT, Wild Type; MUT, Mutant Type.

In many diseases, cytoplasmic lncRNAs competed for binding with miRNAs as competitive endogenous RNA (ceRNA).^24^ Then, the authors collected a number of miRNAs that can bind to SNHG1 highly (Fig. 2A). The prognosis of these miRNAs was predicted by Kaplan-Meier analysis in KM plotter database, which showed that the low expression of miR-21-5p, miR-137-3p, miR-194-5p, and miR-4735-3p was correlated with poor survival (Fig. 2B). This suggested that these four miRNAs might be important miRNAs and mediated the carcinogenic effect of SNHG1. To figure out that, the authors performed q-PCR assays to detect miR-21-5p, miR-137-3p, miR-194-5p, and miR-4735-3p expressions in BC cells after SNHG1 overexpression. And SNHG1 overexpression significantly downregulated miR-137-3p compared to the empty vector group (Fig. 2C). In contrast, the expression of miR-137-3p was stimulated by silencing SNHG1 compared to the NC group in J827 cells (Fig. 2D). Then miR-137-3p expression in BC cells was detected by q-PCR assay. It was indicated that compared to immortalized SV-HUC1 cells, the expression of miR-137-3p in BC cells was higher (Fig. 2E). To further explore the relationship between SNHG1 and miR-137-3p, the authors first overexpressed miR-137-3p in J827 cells and knocked it down in 5637 cells (Fig. 2F). Then, using Ago2-RIP assay after transfection of miR-137-3p mimics, anti-Ago2 antibodies enriched SNHG1 more significantly than the NC group (Fig. 2G). Additionally, the binding sites of SNHG1 on miR-137-3p were predicted. Compared with NC mimics, miR-137-3p mimics reduced Wild-type (Wt) SNHG1 luciferase activity, however, they had no effect on Mutant (Mut) SNHG1 (Fig. 2H). The results indicated that SNHG1 could absorb miR-137-3p.

### SNHG1 acted as a ceRNA to upregulate EZH2 in the cytoplasm

**Fig. 3:**
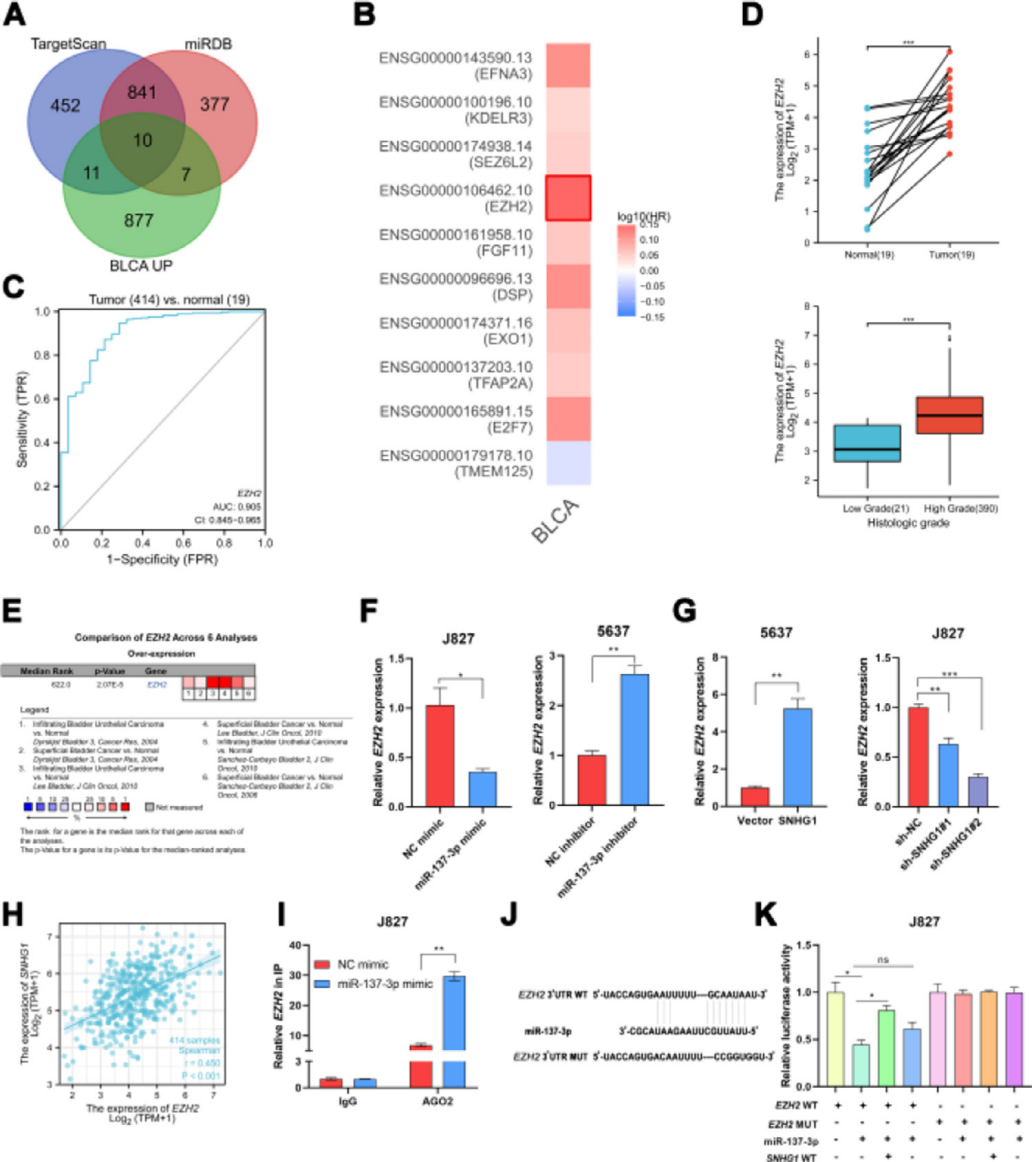
SNHG1 upregulated EZH2 through sponging miR-137-3p in the cytoplasm. A, Prediction of downstream genes of miR-137-3p in the intersection of TargetScan database, miRDB database and upregulated genes in BLCA. B, The prognostic significance of ten up-regulated genes. C, The ROC curve analysis of EZH2. D, The expression of EZH2 in BLCA data from TCGA. E, The expression of EZH2 in BC-related datasets from Oncomine. F, The expression of EZH2 in J827 cells with transfection of mimics or inhibitors. G, EZH2 expression in 5637 cells with SNHG1 overexpression or in J827 cells with SNHG1 knockdown. H, Correlation of EZH2 and SNHG1, the data were analyzed from BC TCGA database. I, Enrichment of EZH2 pulled down by AGO2 antibodies in J827 cells with mimic transfection. J, Prediction of binding sites of EZH2 on miR-137-3p. K, The luciferase activity in J827 cells co-transfected with SNHG1 plasmids, EZH2 plasmids and mimics. Statistical significance was assessed using t test for two groups or one-way ANOVA for multiple groups. *p < 0.05; **p < 0.01. BLCA, Bladder Urothelial Carcinoma; WT, Wild Type; MUT, Mutant Type.

In order to explore the potential target genes of miR-137-3p, the authors first used two bioinformatics databases (TargetScan and miRDB) to predict the potential interaction between miR-137-3p and mRNA, and ten up-regulated mRNAs were found in BLCA, including TMEM125, E2F7, TFAP2A, EXO1, DSP, FGF11, EZH2, SEZ6L2, KDELR3 and EFNA3 (Fig. 3A). Then, by searching GEPIA2, the authors found that only EZH2 was associated with shorter disease-free survival of BLCA patients (Fig. 3B). And ROC curve analysis showed that the AUC of EZH2 was 0.905, which indicated that EZH2 had a good diagnostic value (Fig. 3C). In addition, compared to normal bladder tissues, the TCGA data displayed that the EZH2 expression was higher in BLCA tissues, and higher in tumors of a high histologic grade than in tumors of low histologic grade (Fig. 3D). And it was found that EZH2 was highly expressed in six BC-related datasets from Oncomine (Fig. 3E). To investigate whether EZH2 was the downstream gene of miR-137-3p, the authors transfected J827 cells with miR-137-3p mimics and transfected 5637 cells with miR-137-3p inhibitors. The results showed that the EZH2 expression could be suppressed by miR-137-3p (Fig. 3F). SNHG1 overexpression prominently enhanced EZH2 expression, while sh-SNHG1#2 noticeably inhibited the expression of EZH2 (Fig. 3G). Besides, SNHG1 was positively correlated with EZH2 in BLCA data from TCGA (Fig. 3H). Furthermore, it was found that miR-137-3p mimics reduced the enrichment of EZH2 pulled down with anti-Ago2 antibodies (Fig. 3I). And compared with NC mimics, Wt EZH2 luciferase activity was decreased by miR-137-3p mimics. Wt SNHG1 could enhance the luciferase activity but had no effect on the Mut EZH2 3′UTR reporter plasmid (Fig. 3K). The above studies illustrated that EZH2 was the target gene of miR-137-3p, and SNHG1 promoted EZH2 expression by absorbing miR-137-3p to promote BC development.

### SNHG1 promoted proliferation, migration, invasion and EMT of BC cells by absorbing miR-137-3p

**Fig. 4:**
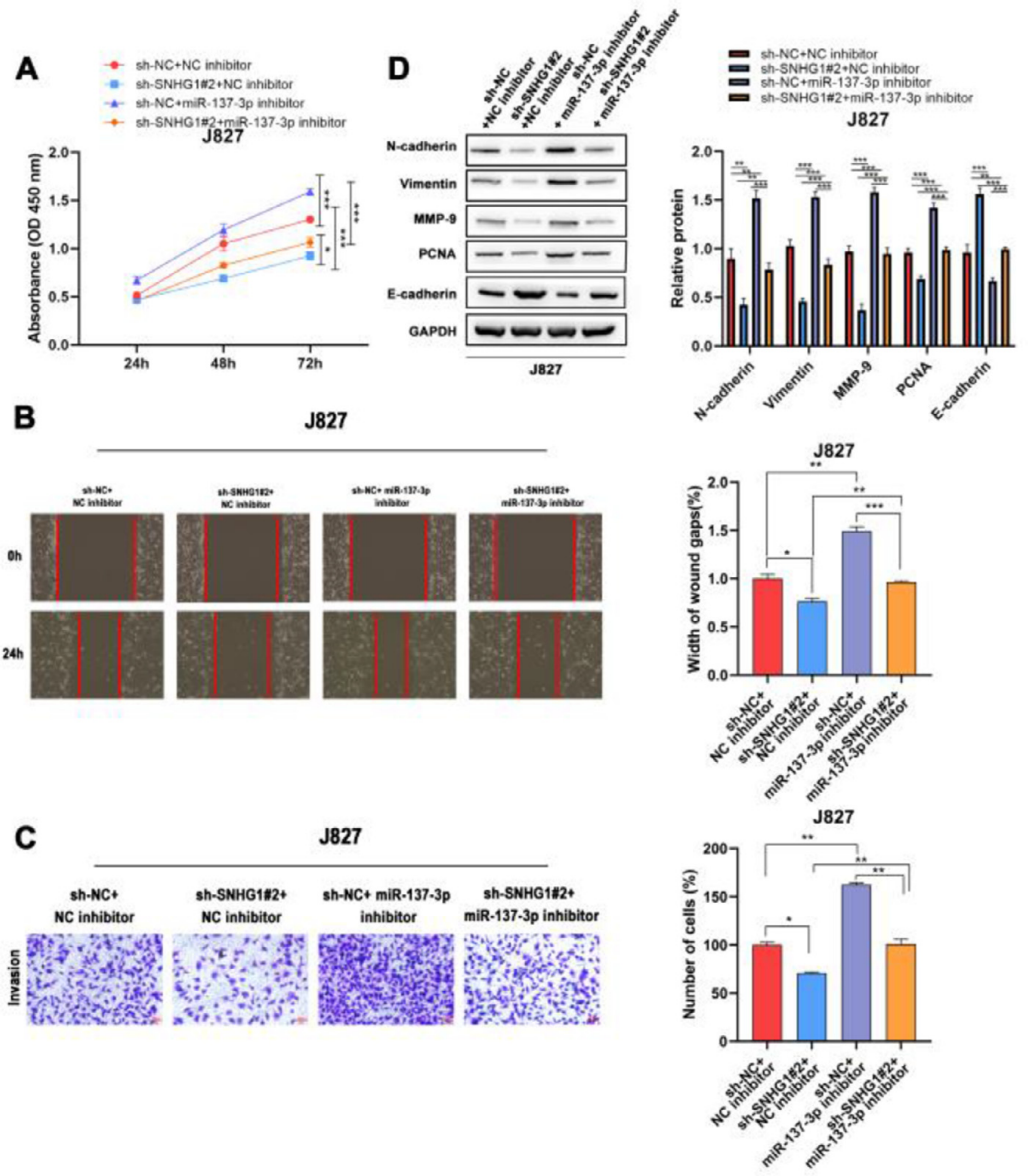
SNHG1 absorbed miR-137-3p to promote BC cell proliferation, migration, invasion and EMT. A, The proliferation of J827 cells was detected with CCK-8. B, The migration of J827 cells was detected with Wound healing assay. C, The invasion of J827 cells was detected with transwell assay D, The protein levels of PCNA, N-cadherin, Vimentin, E-cadherin and MMP-9 in J827 cells was detected with Western blot. Statistical significance was assessed using one-way ANOVA for multiple groups. *p < 0.05; **p < 0.01.

To investigate the effects of SNHG1 on the regulation of cell functions, the CCK8 assay was used to detect the proliferation of J827 cells. The results showed that SNHG1 knockdown inhibited cell proliferation, while the inhibitory effect could be partially blocked by miR-137-3p inhibitors (Fig. 4A). Wound healing and transwell assays showed that sh-SNHG1#2 inhibited the migration and invasion, which could be blocked by miR-137-3p inhibitors (Fig. 4B-C). Additionally, Western blot showed that SNHG1 silencing decreased the expression of PCNA, N-cadherin, Vimentin, and MMP-9, but increased the E-cadherin expression, which could be rescued by miR-137-3p knockdown (Fig. 4D). The results indicated that SNHG1 promoted the growth and tumorigenesis of BC cells by binding with miR-137-3p.

### SNHG1 participated in the transcriptional repression of KLF2 through its interaction with EZH2

**Fig. 5:**
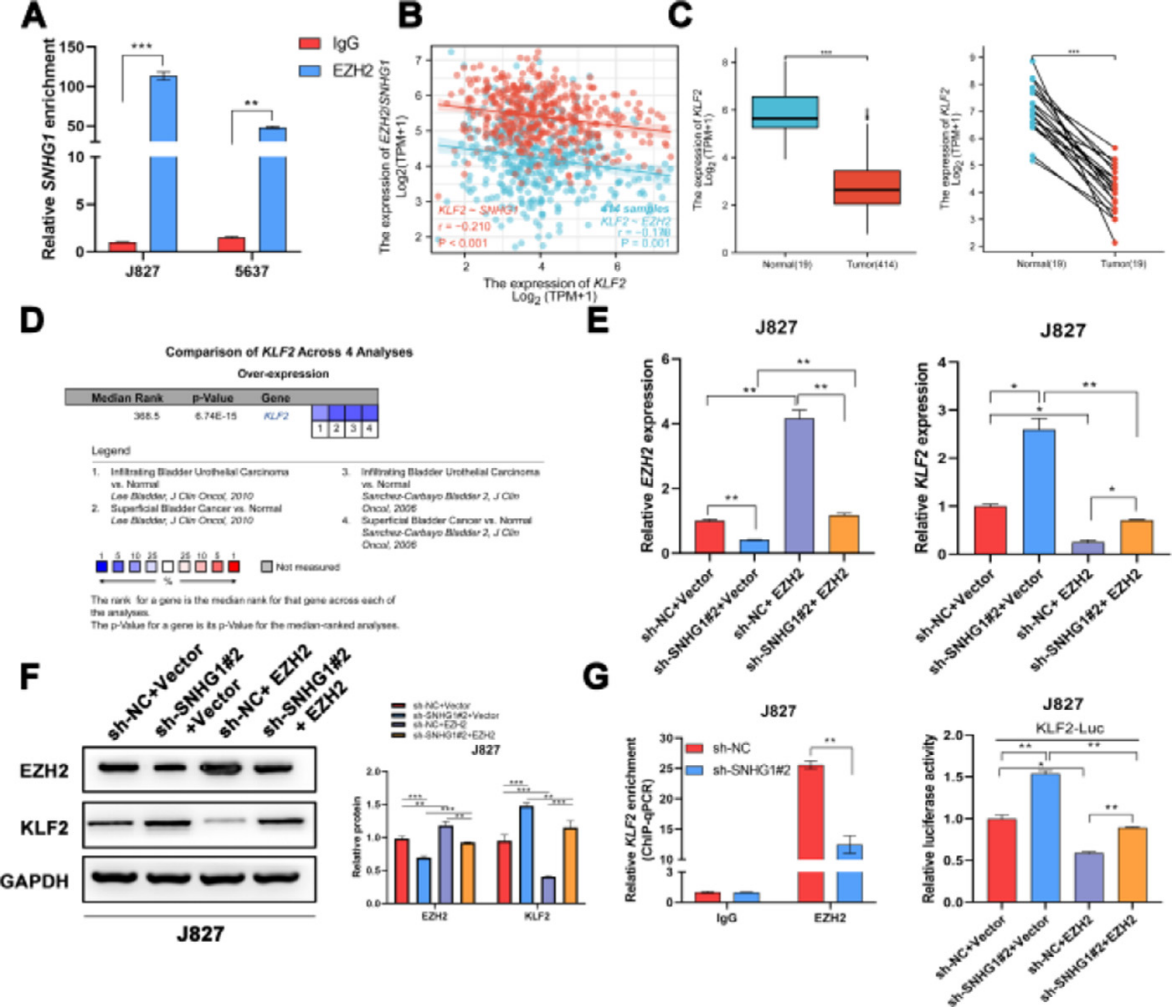
SNHG1 was involved in the transcriptional repression of KLF2 through its interaction with EZH2. A, SNHG1 was pulled down by EZH2 antibody. B, Correlation of KLF2 and SNHG1/EZH2, the data were analyzed from BC TCGA database. C, KLF2 expression in BLCA data from TCGA (left) and fresh frozen specimen (right). D, KLF2 expression in BC-related datasets from Oncomine. E, After SNHG1 knockdown or EZH2 overexpression, the mRNA levels of EZH2 and KLF2 in J827 cells were detected by qPCR assay. F, After SNHG1 knockdown or EZH2 overexpression, the protein levels of EZH2 and KLF2 in J827 cells were detected by Western blot assay. G, The enrichment of KLF2 promoter pulled down by EZH2 antibodies in J827 cells with SNHG1 silencing. H, The transcriptional activity of KLF2 promoter in J827 cells with SNHG1 knockdown or EZH2 overexpression. Statistical significance was assessed using t test for two groups or one-way ANOVA for multiple groups. *p < 0.05; **p < 0.01; ***p < 0.001.

It was reported that EZH2 participated in multiple tumorigeneses through epigenetic repression of its targets.^25^ Here, the authors successfully pulled down SNHG1 with EZH2 antibody (Fig. 5A). EZH2 was reported to transcriptionally inhibit the expression of KLF2.^26^ Besides, KLF2, was negatively correlated with SNHG1 and EZH2 in BLCA data from TCGA (Fig. 5B). Compared to normal bladder tissues, KLF2 was lowly expressed in BLCA tissues (Fig. 5C). In addition, the authors found lowly expressed KLF2 in four BLCA-related datasets from Oncomine (Fig. 5D). To explore whether SNHG1 and EZH2 could regulate KLF2 expression, the authors transfected J827 cells with sh-SNHG1#2 or EZH2. The results of q-PCR and Western blot showed that silencing SNHG1 downregulated EZH2, and KLF2 was upregulated in J827 cells. Transfection of p-CMV-HA-EZH2 could effectively enhance EZH2 expression and obviously, impede KLF2 expression (Fig. 5E‒F). Furthermore, the authors carried out a ChIP assay, and it was found that the enrichment of the KLF2 promoter fragment pulled down by EZH2 antibodies was extremely low in SNHG1 silenced cells (Fig. 5G). In addition, sh-SNHG1#2 promoted the transcriptional activity of KLF2 promoter detected by luciferase reporter gene assay, while the overexpression of EZH2 inversely reduced the transcriptional activity of KLF2 promoter (Fig. 5H). These results suggested that SNHG1 participated in the transcriptional repression of KLF2 through the recruitment of EZH2.

### SNHG1 knockdown inhibited the tumorigenesis in vivo

**Fig. 6:**
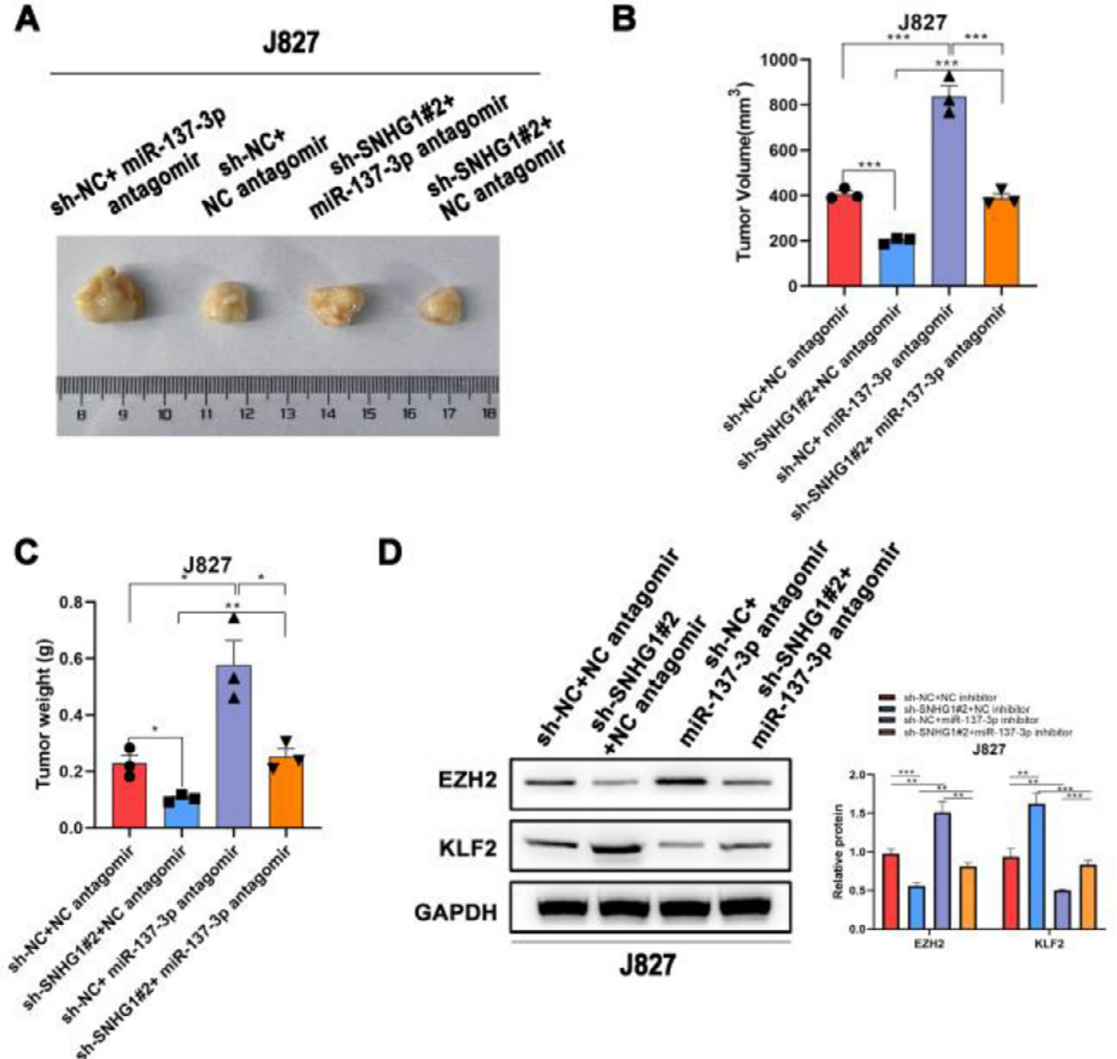
SNHG1 knockdown inhibited the tumorigenesis *in vivo*. A‒C, J827 cells were stably transfected with sh-SNHG1#2 vectors, empty vectors, miR-137-3p antagomir or NC antagomir, and subcutaneously injected into nude mice (n = 5). There were four groups, including Vector +NC antagomir, sh-SNHG1#2 + NC antagomir, Vector + miR-137-3p antagomir, sh-SNHG1#2 + miR-137-3p antagomir. Representative images (A), tumor volume growth curves (B) and tumor weight (C). D, The protein expression of EZH2 and KLF2 in subcutaneous tumor tissues of different groups. Statistical significance was assessed one-way ANOVA for multiple groups. *p < 0.05; **p < 0.01.

To investigate the effects of SNHG1 on the carcinogenesis of BC cells *in vivo*, the authors subsequently injected J827 cells stably transfected with sh-SNHG1#2 into nude mice to establish a tumor growth model. Compared with the NC group, sh-SNHG1#2 dramatically decreased the tumor volume and weight, which were obviously increased in the sh-NC+miR-137-3p antagomir group (Fig. 6A‒C). And Western blot was carried out to detect EZH2 and KLF2 expression in different groups. It was revealed that SNHG1 silencing reduced EZH2 expression and promoted KLF2 expression. This phenomenon was reversed by miR-137-3p antagomir (Fig. 6D). The results above indicated that SNHG1 promoted EZH2 expression and suppressed KLF2 transcription, thus promoting the growth and tumorigenesis of BC cells *in vivo*.

## Discussion

The present study demonstrated a high expression of SNHG1 in BC, and up-regulation of SNHG1 was closely related to the poor survival. Functional investigation showed that SNHG1 promoted tumor proliferation, migration, and tumorigenesis. Mechanistically, SNHG1 sponged miR-137-3p to promote the expression of EZH2 in the cytoplasm. And in the nucleus, SNHG1 recruited EZH2 to suppress KLF2 expression. These results illustrated that SNHG1 played an oncogenic role in BC.

LncRNAs are a special group of RNAs that do not encode proteins.^27^ They are involved in gene regulation at the transcriptional and post-transcriptional levels in different environments.^28^ It is reported that SNHG1 is aberrantly expressed in a variety of diseases, and plays a crucial role in disease development.^29^ SNHG1 activated the Wnt/β-catenin pathway to promote cell growth in Acute Myeloid Leukemia (AML).^30^ In addition, SNHG1 regulated the translation pathway of E-cadherin by reducing the expression of hnRNPL to promote the metastasis of cancer cells.^31^ SNHG1 also facilitated proliferation and metastasis of Ovarian Cancer (OC) by activating Akt signaling pathway.^32^ And SNHG1 contributed to the enhancement of drug resistance in tumor cells.^33^ Previous studies also reported high expression of SNHG1 in BC, which promoted BC cell invasion.^34^ In this study, the high expression of SNHG1 in BC was confirmed again by bioinformatics techniques and clinical samples. And the high expression of SNHG1 was strongly correlated with poor outcomes. The results suggested that SNHG1 had a crucial role in BC development.

The lncRNA-miRNA-mRNA network has become one of the research hotspots because of its indispensable role in a variety of cancers.^35^, ^36^, ^37^ miRNAs are also a special class of ncRNAs, which consist of about 20 nucleotides.^38^ And miRNAs regulate downstream genes by binding to the 3 'untranslated region.^39^ Interestingly, lncRNAs can eliminate the inhibition of downstream targets by competitively binding target miRNAs.^36^ Several studies revealed that, in the cytoplasm, lncRNAs acted as ceRNAs and adsorbed target miRNAs to regulate the expression of downstream genes.^40^ For instance, LINC00675 inhibited hepatocellular carcinoma metastasis by reducing miR-942-5p and promoting GFI1 expression.^24^ LncRNA UCA1 promoted MYO6 expression through adsorbing miR-143, thus promoting the proliferation of prostate cancer (PCa).^41^ Here, miR-137-3p was discovered as the target miRNA of SNHG1. And miR-137-3p silencing can promote BC cell proliferation, migration, and EMT. It was demonstrated that SNHG1 absorbed miR-137-3p to promote EZH2 expression, thus promoting BC progression.

Many studies have shown that some lncRNAs could recruit EZH2 to participate in the transcriptional repression of target genes in multiple cancers and diseases.^25^^,^^42^^,^^43^ Here, it was found that SNHG1/EZH2 was negatively correlated with KLF2. Numerous studies have shown that the expression of KLF2 is reduced in various cancers, and it plays a role in inhibiting tumor cell proliferation and inducing apoptosis.^44^^,^^45^ These suggested that SNHG1 directly interacted with EZH2 to modulate the transcriptional activity of KLF2.

In conclusion, the present results illustrated that SNHG1 acted as an oncogenic lncRNA and was correlated with poor survival of BC patients. And SNHG1 displayed different regulatory mechanisms in a diverse subcellular environment to promote the proliferation and tumorigenesis of BC cells. In the cytoplasm, SNHG1 competitively bound miR-137-3p to promote EZH2 expression. And in the nucleus, SNHG1 was involved in the epigenetic repression of KLF2 through the recruitment of EZH2. The present study has elucidated the oncogenic role of SNHG1 and provides a potential therapeutic biomarker for BC treatment.

## Authors’ contributions

Jie Min, Jiaxing Ma, Qi Wang and Dexin Yu conceived the project, designed and performed the experiments, and analyzed the data. Qi Wang and Dexin Yu wrote the manuscript.

## Funding

This work was supported by the Anhui Medical University Natural Science Foundation (grant number 2020xkj194); Clinical Research Cultivation Program of The Second Affiliated Hospital of Anhui Medical University (grant number 2020LCYB11) and Natural Science Fund for Colleges and Universities in Anhui Province (grant number KJ2021A0312).

## Conflicts of interest

The authors declare no conflicts of interest.

## Appendix Supplementary materials

Image, application 1
