# Supplementary material for: Long non-coding RNA SNHG1 promotes bladder cancer progression by upregulating EZH2 and repressing KLF2 transcription
Source: Clinics (Sao Paulo). 2022 Sep 7;77:100081. doi: 10.1016/j.clinsp.2022.100081 (PMC9468346; doi:10.1016/j.clinsp.2022.100081)
Supplement: Supplementary file 1 [file mmc1.pdf]

CLINICS-2021-3716 – Supplementary Material

Supplementary Table 1 Sequences of PCR primers used in this study.

**SNHG1**	Forward (5'-3')	TGTCTGTGTTCACTCCAGGC
	Reverse (5'-3')	TGGCTCCCAGTGTCTTAATCTG
**miR-21-5p**	Forward (5'-3')	TAGCTTATCAGACTGATGTTGA
	Reverse (5'-3')	AGTGCGTGTCGTGG
**miR-137-3p**	Forward (5'-3')	GAT TTA TGG TCC CGG TCA AG
	Reverse (5'-3')	AAT ACC CGT CAC CGA AGA GA
**miR-194-5p**	Forward (5'-3')	AGT GTG ACG TTG ACA TCC GT
	Reverse (5'-3')	GCA GCT CAG TAA CAG TCC GC
**miR-4735-3p**	Forward (5'-3')	GAAGGTGCTCAAACCAGACAT
	Reverse (5'-3')	CTCTACAGCTATATTGCCAGCCA
**EZH2**	Forward (5'-3')	TCCCGCTGAGGATGTGGATA
	Reverse (5'-3')	ATCACACAAGGGCACGAACT
**KLF2**	Forward (5'-3')	GTCCTTCTCCACTTTCGCCA
	Reverse (5'-3')	ACAGGATGAAGTCCAGCACG
**GAPDH**	Forward (5'-3')	GTCAAGGCTGAGAACGGGAA
	Reverse (5'-3')	AAATGAGCCCCAGCCTTCTC
**U6**	Forward (5'-3')	CTCGCTTCGGCAGCACA
	Reverse (5'-3')	AACGCTTCACGAATTTGCGT

Supplementary Table 2 Sequences of shRNA against specific targets.

sh-SNHG1#1	5'-3'	GGGTATTTCAGATGTACCTTA
sh-SNHG1#2	5'-3'	GGTTTGCTGTGTATCACATTT
